# The Neutron Imaging Instrument CONRAD—Post-Operational Review

**DOI:** 10.3390/jimaging7010011

**Published:** 2021-01-19

**Authors:** Nikolay Kardjilov, Ingo Manke, André Hilger, Tobias Arlt, Robert Bradbury, Henning Markötter, Robin Woracek, Markus Strobl, Wolfgang Treimer, John Banhart

**Affiliations:** 1 Helmholtz-Zentrum-Berlin, D-14109 Berlin, Germany; manke@helmholtz-berlin.de (I.M.); hilger@helmholtz-berlin.de (A.H.); tobias.arlt@helmholtz-berlin.de (T.A.); banhart@helmholtz-berlin.de (J.B.); 2 Fakultät III Prozesswissenschaften, Technische Universität Berlin, D-10623 Berlin, Germany; robert.bradbury@tu-berlin.de; 3 Bundesanstalt für Materialforschung und-prüfung, D-12205 Berlin, Germany; henning.markoetter@bam.de; 4 European Spallation Source, SE-221 00 Lund, Sweden; robin.woracek@ess.eu; 5 Paul-Scherrer-Institut, CH-5232 Villigen, Switzerland; markus.strobl@psi.ch; 6 Beuth Hochschule für Technik Berlin, D-13353 Berlin, Germany; wolfgang.treimer@beuth-hochschule.de

**Keywords:** neutron imaging, neutron scattering, neutron instrument, tomography

## Abstract

The neutron imaging instrument CONRAD was operated as a part of the user program of the research reactor BER-II at Helmholtz-Zentrum Berlin (HZB) from 2005 to 2020. The instrument was designed to use the neutron flux from the cold source of the reactor, transported by a curved neutron guide. The pure cold neutron spectrum provided a great advantage in the use of different neutron optical components such as focusing lenses and guides, solid-state polarizers, monochromators and phase gratings. The flexible setup of the instrument allowed for implementation of new methods including wavelength-selective, dark-field, phase-contrast and imaging with polarized neutrons. In summary, these developments helped to attract a large number of scientists and industrial customers, who were introduced to neutron imaging and subsequently contributed to the expansion of the neutron imaging community.

## 1. Chronology

2004–2010: The imaging facility V7 (CONRAD-1) at the 10 MW BER II medium-flux research reactor was designed in 2004 and constructed in 2005 as an instrument supporting the materials research activities at the former Hahn-Meitner-Institute (HMI) [1,2]. At that time, CONRAD-1 was situated at the neutron guide NL-1B (m = 1.2, ^58^Ni coating) with a characteristic wavelength of 2.2 Å [3]. This neutron guide also served another two instruments in front of CONRAD-1: the reflectometer V14 and the triple-axis spectrometer V2 FLEX. The use of a neutron guide for an imaging instrument was challenging for the project since only feasibility tests and simulations of such geometry had been performed up to that time [4]. CONRAD-1 was one of the first user imaging instruments that used a curved neutron guide for neutron transport. For this reason, Monte Carlo simulations of the guide system, as well as the instrument design, were performed to optimize the instrument parameters [1]. The available distance of 5 m behind the neutron guide was quite short for a collimation path, which resulted in a beam size limited to approximately 10 cm × 10 cm at the detector position, due to the neutron divergence provided by the guide. The small size was a competitive disadvantage in comparison with other existing facilities around the world, where beams double the size and more were available for conventional imaging. This was, however, a motivation for concentrating on the development of novel methods that benefit from the cold neutron beam and the low background at the instrument [5,6]. The implementation of these new techniques as standard instrument options helped to expand the imaging capabilities of the beamline, allowing for imaging with polarized neutrons [7,8,9], Bragg-edge mapping [10,11,12,13], high-resolution neutron imaging [14] and grating interferometry [15,16]. These methods were offered to the user community as tools to help address scientific problems over a broad range of topics, such as superconductivity [17], materials research [18,19], life sciences [20,21], cultural heritage and paleontology [22,23]. Industrial applications, including fuel cell [24,25] and battery research [26,27,28], have also been fostered by these increased capabilities, which further helped to increase and improve the scientific output of the facility and to attract new users.

In 2007, an X-ray imaging Lab (MicroCT Lab) was established, which allowed for imaging experiments complementary to neutron imaging. The MicroCT scanner has been used extensively by users for feasibility tests and small projects. This has helped to build a bridge to neutron imaging for many users from the X-ray community. Today, the MicroCT lab is well established with high user demand.

2009–2012 (Instrument Upgrade): In 2009 CONRAD-1 the platform above the instrument was enlarged and used as the instrument “control room”. In addition, the shielding of the collimation upstream of the neutron guide (made of concrete) was replaced by a new one using an improved design (sandwich of 5 mm B_4_C plates and 10 cm Pb). As a result, the dose rate around the facility was reduced and the space on the control platform was enlarged.

**Figure 1:**
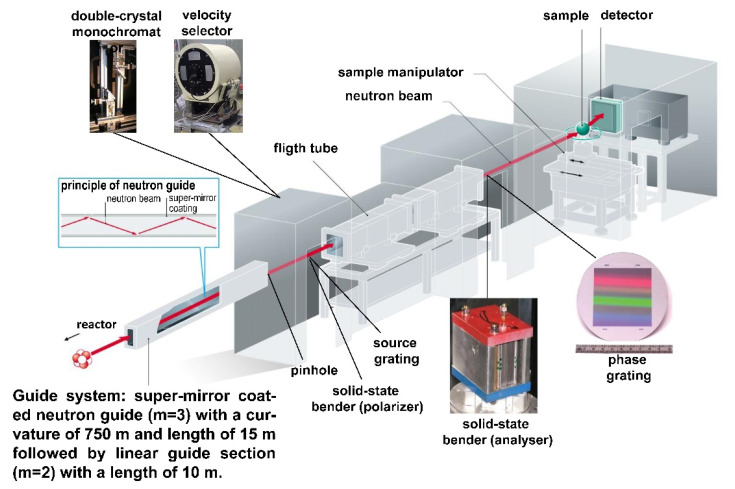
Layout of the CONRAD-2 Instrument.

During the cold neutron instrumentation upgrade at BER-II from October 2010 to October 2012, the cold neutron source was replaced and the neutron guide system serving the instruments in neutron guide hall I was completely redesigned and updated. The CONRAD instrument (after the upgrade renamed CONRAD-2) was moved to a new location in the facility that allowed for a longer collimation path of 10 m. The old neutron guides (m = 1.2) were also exchanged for new supermirror guides (m = 2), which increased the beam divergence. These modifications to the instrument improved the efficiency of the neutron transport and increased the available beam size. Additionally, the curvature of the guide was increased by reducing its radius from R = 3000 m to R = 750 m in order to increase the distance from the shielding of the neighboring instrument and to provide a more spacious experimental and user environment, Figure 1 [29].

2012–2019: After the successfully completed upgrade, the neutron intensity at the end of the guide (at the pinhole position) was 2.7 × 10^9^ n/cm^2^s, which was an order of magnitude higher than before the upgrade. The measured intensity at the detector position (a distance 10 m from the pinhole) was 2.4 × 10^7^ n/cm^2^s for an L/D = 350, resulting in a gain of 2.4 in comparison with the same instrument configuration from before the upgrade. The obtained beam size increased to 30 cm × 30 cm, allowing for investigations of larger samples [29].

The instrument parameters and options are given in detail in Table 1.

## 2. Scientific Case

V7 has widely been recognized as a versatile and flexible instrument for innovative cold neutron imaging and has made seminal contributions to the development of new methods by exploiting different contrast mechanisms for imaging [22,23,30]. The reason for the success in the development of instrument capabilities was the flexibility of the facility, which permitted very fast changes of the instrument configurations and allowed for non-standard experiments. The ability to perform complementary experiments with the laboratory X-ray tomographic scanner (µ-CT Lab) offered the opportunity to study samples at different contrast levels and spatial resolution scales.

CONRAD-2 was well suited not only for attenuation contrast radiography and tomography, frequently used in industrial applications, but also for wavelength-selective measurements due to the installed double-crystal monochromator [11] and velocity selector. Solid-state polarizers [8] and polarized ^3^He filters [31] were used for imaging with polarized neutrons. A phase grating setup [32] could be used for grating interferometry experiments, where phase contrast and dark-field imaging were used to obtain spatially resolved information about the microstructure of the materials in question [16] or their magnetic properties [15]. The instrument also had a prototype of a high-resolution detector which could provide images of samples with a pixel size down to 6.5 µm at reasonable exposure times [14]. We will now highlight below some of the most important instrument modalities, with examples from different research fields, that made use of the CONRAD-2 instrument.

### 2.1. Attenuation Contrast Imaging Using a Direct Mode

**Figure 2:**
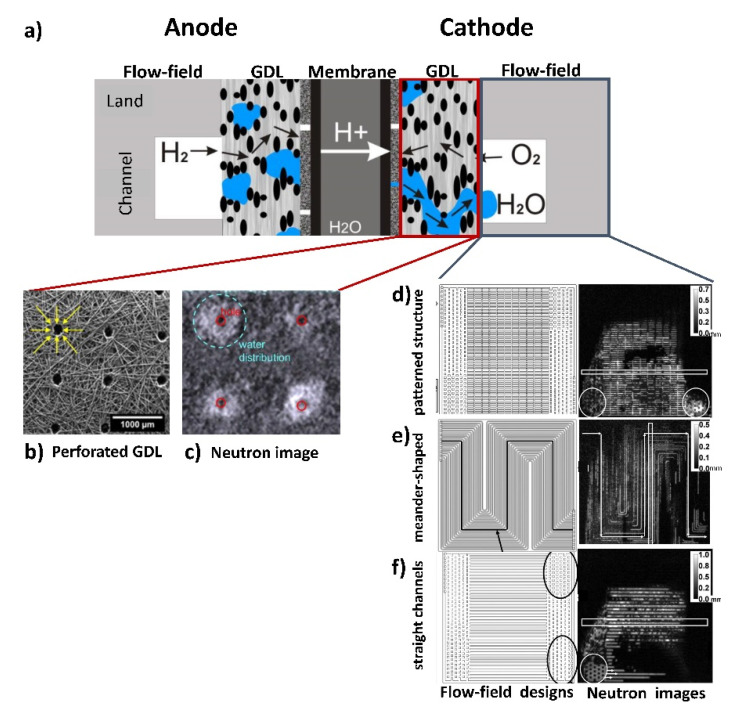
Component optimization of Polymer Electrolyte Membrane Fuel Cell (PEMFC): (**a**) Scheme of PEMFC. (**b**) Perforated Gas-Diffusion-Layer (GDL) hydrophobic material improves the water drainage. (**c**) Neutron tomographic slice shows a failure of the GDL material where the water is detected in the hydrophobic matrix (white areas) due to overheating at laser drilling of the holes. (**d**–**f**) Dynamic performance of three different flow field designs. On the left: Design drawings of the cathode flow fields (from top to bottom: patterned, meandering flow field, straight channels). On the right: The current water distribution in the investigated flow fields visualized by dynamic neutron imaging. Water thickness is given in mm [43,44].

Fuel cell research: The enhanced contrast of water in the presence of metal components, provided by neutron imaging allowed for in-situ and operando investigations of the water distribution in operating low-temperature fuel cells [25,33,34,35,36,37,38,39,40]. Through use of this technique, very small amounts of water (min 10 µm thickness) can be visualized and analyzed [41]. Dynamic neutron imaging helps to study water transfer processes in single and multiple fuel cell stacks with frame rates of 6 to 30 frames per minute. Tomographic investigations allow for three-dimensional visualization and analysis of water distributions in such stacks [42], Figure 2.

**Figure 3:**
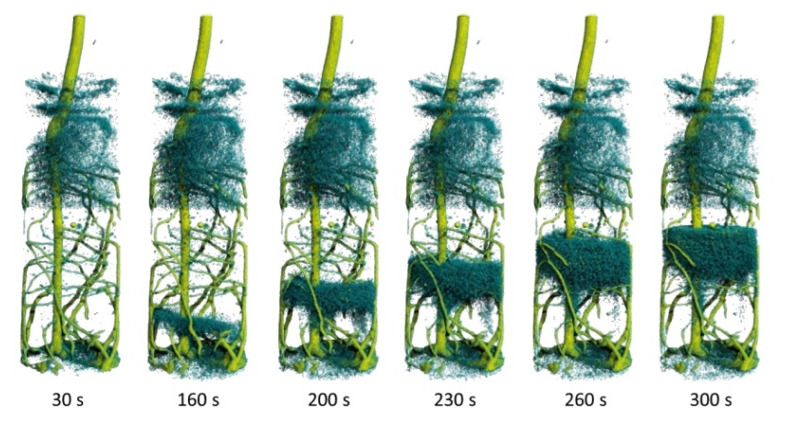
Visualization of the water uptake by the root system of a lupine by dynamic neutron tomography after the injection of 4 mL deuterated water (D_2_O) through the bottom. The time series (30 s ≤ t ≤ 300 s) shows the ascending front of water (H_2_O) moving upwards as it is being displaced by the injected deuterated water. The repetition time for the tomograms is just 10 s [48]. Copyright: Christian Tötzke (University of Potsdam, Germany), published in [48]. The image is included in the article’s Creative Commons license: http://creativecommons.org/licenses/by/4.0/.

Life science: Water transport in plants and the root-soil interaction processes can be visualized by dynamic neutron radiography using D_2_O as a tracer. [45,46]. The neutrons can distinguish between different isotopes of one element and show significant changes in the transmission e.g., light (H_2_O) and heavy (D_2_O) water results in low and high beam transmission respectively. In this way, parameters such as the velocity of water uptake and the reaction to toxic atmospheres or soil conditions have been investigated [47,48,49], Figure 3.

**Figure 4:**
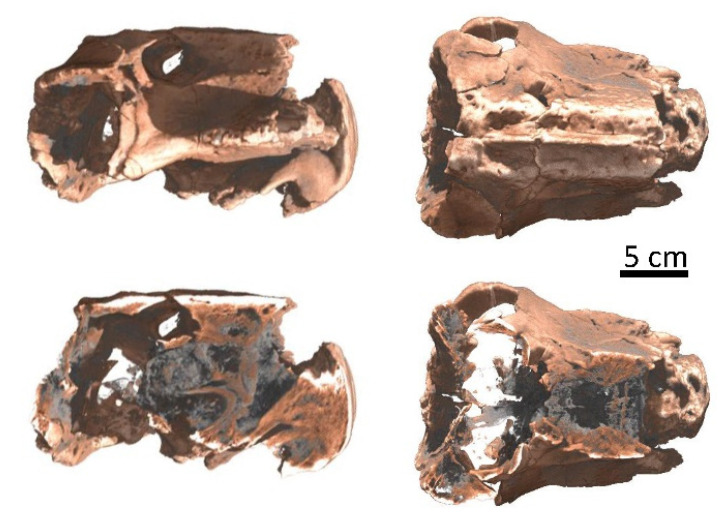
3D representation of a skull of Lystrosaurus declivis (Therapsida, Anomodontia) from the Lower Triassic from South Africa obtained by neutron tomography investigation. The digital processing of the data allows for sections in the skull revealing a complexly constructed nasal cavity, which provides evidence that Lystrosaurus was already endothermic. The endothermic metabolism allowed Lystrosaurus to tolerate high ambient temperature fluctuations [56].

Archaeology, paleontology and geology: The high penetration power of the neutron beam through rocks and metals makes neutron tomography a unique tool for non-destructive investigations of a broad range of samples, ranging from metal objects such as historical weapons [50,51,52] or ancient sculptures [53] to fossils [54,55,56] and geological samples [57], Figure 4.

**Figure 5:**
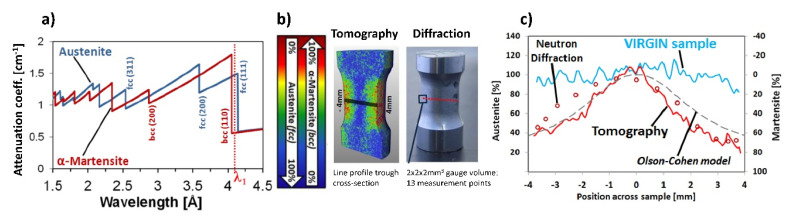
Wavelength-selective imaging and Bragg edge analysis. (**a**) The selected wavelength λ_1_ provides a significant difference between the theoretical attenuation coefficients of austenite and α-martensite. (**b**) Tomography experiment at wavelength λ_1_ helps to obtain the 3D distribution of phase fractions inside a sample subjected to torsional loading. Large plastic deformation close to the surface of the sample has led to the formation of martensite. (**c**) The phase fractions obtained from the tomography experiment along a line profile were compared with data from standard neutron diffraction measurement and with the theoretical α-martensite phase evolution using the Olson–Cohen model [13].

Wavelength-selective imaging: A single wavelength can be selected from the polychromatic neutron beam through use of the double crystal setup, over a range from 1.5 Å to 6.0 Å, with a wavelength resolution of approximately 1–3% [58]. The monochromatic neutron beams selected in this way, and the possibility for continuous wavelength scans, allowed for a broad range of applications where the crystallographic related properties of the materials were probed e.g., residual stress accumulation and annealing [12], analysis of fatigue [13] and optimization of welding techniques (e.g., Friction Steer Welding) [59] as well as various industrial inspection procedures. An important feature of this method is its sensitivity to material phase separation, where the neutron wavelength is selected to be between Bragg edges of two material phases (e.g., γ- and α–ferrite) [60]. A combination of this technique with tomography allows for a determination of local phase fractions in multiphase crystalline materials [13], Figure 5.

**Figure 6:**
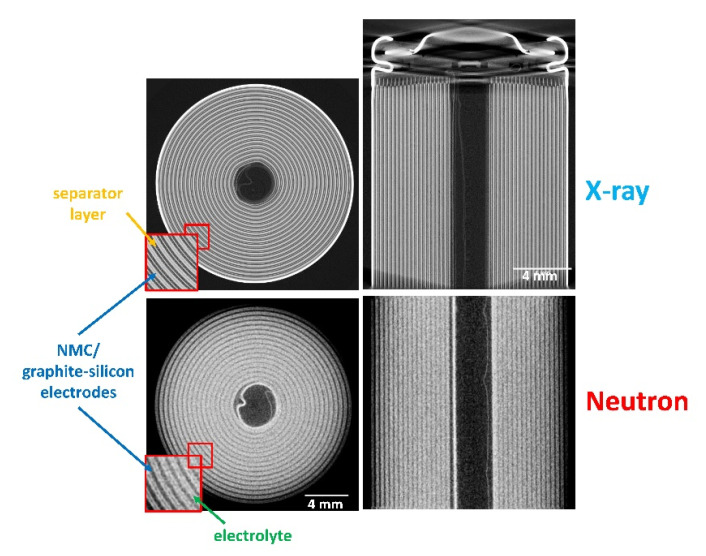
Comparison of X-ray and high-resolution neutron tomography with the same pixel size (6.5 µm) of a Li-ion cell (LG 18650 MJ1) with NMC cathode and new graphite-silicon anode for a high capacity of 3500 mAh. Image courtesy: Ralf Ziesche, UCL, UK.

High resolution imaging: Application areas include innovative microcellular materials such as metal and polyester foam structures, porous materials such as Membrane Electrode Assemblies (MEA) or gas diffusion layers, the latter two being crucial components of fuel cells. The high penetration depth of a neutron beam in metals combined with the high-sensitivity to Li and hydrogen makes high-resolution imaging an ideal method for visualization of lithiation processes and electrolyte distribution in Li-ion batteries [26,27,28], Figure 6.

Time-resolved studies: Stroboscopic techniques allow for the observation of fast periodic phenomena with the imaging power of neutrons. Simple attenuation contrast imaging of fast processes (e.g., water uptake in rocks) has been demonstrated to be feasible in the range of 20 fps using a high-speed sCMOS camera [61]. The on-the-fly tomography technique [49] allowed for investigation of dynamic processes in 3-D with time resolutions better than 1 min, as shown in Figure 3. Imaging of alternating magnetic fields, however, could be developed into a versatile technique without competition due to the unique properties of neutron interactions. A feasibility test has allowed time resolved imaging of a magnetic field with 105 fps (using an MCP detector) [62].

### 2.2. Beyond Attenuation Contrast, Various Scientifically Promising Fields Have Emerged

**Figure 7:**
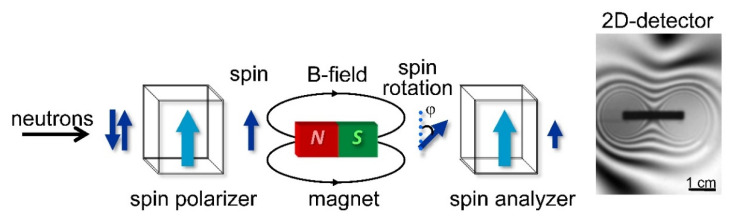
Imaging with polarized neutrons: The spin-polarizer filter accepts only one spin component of the incoming neutrons. The polarized beam then passes the magnetic field of a sample during which the neutron spin rotates by an angle φ. Depending on the resultant rotation angle φ, the transmission through analyzer ranges from 0 to 1. This gives rise to a grey-scale image after measurement by the 2-D detector. In the example given on the right, the field distribution around a magnet is visible [7].

Imaging with polarized neutrons: Polarized neutron imaging utilizes a spin polarizer-analyzer arrangement as shown in Figure 7. This arrangement helps to convert the precession angle of the neutron spin, accumulated while passing through a magnetic field, to image contrast. As a technique, it has some tantalizing prospects for the future study of magnetic phenomena throughout science and technology, including optimization of high-temperature superconducting materials by visualization and analysis of trapped magnetic flux in the bulk of superconductors at different temperatures [17], studies related to the skin effect in conductors [63], and phase mapping of ferro-to-paramagnetic transitions in bulk ferromagnets [64], Figure 7. In some cases, the method allows for quantification of magnetic fields and can also be extended to three dimensions in analogy with standard tomography. To achieve this, the development of advanced algorithms for tomographic reconstruction of complex magnetic vector fields has been successfully achieved [65].

**Figure 8:**
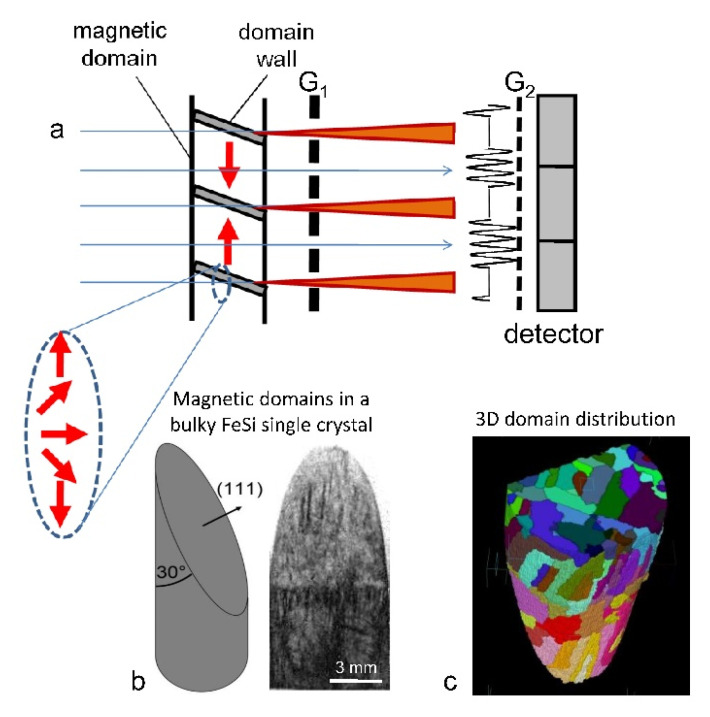
Grating interferometry: A partially coherent neutron beam transmits through the sample and passes through the phase grating G_1_ resulting in an interference pattern. (**a**) Refraction at domain walls decreases locally the amplitude in the interference pattern. (**b**) The position sensitive mapping of the amplitude (dark-field imaging) of a bulky monocrystalline FeSi sample helps to visualize the domain walls as dark lines. (**c**) The magnetic domain structure of a bulk FeSi single crystal can be visualized in 3-D. The color map represents domains of different orientation [15].

Grating interferometry uses a partially coherent neutron beam which, after interaction with the sample, passes through a phase grating G_1_ which produces an interference pattern, Figure 8. The pattern is analyzed by a second grating G_2_, allowing detection of angular beam deflections due to refraction and small-angular scattering. The scattering reduces the amplitude of the interference pattern which can be mapped by a position sensitive detector helping to characterize material heterogeneities on the scale of 0.1 µm to 10 µm [66]. Refraction at the magnetic domain walls can be used to visualize magnetic domains. Using tomographic reconstruction, a 3-D domain network can be analyzed and studied under different external conditions, e.g., varying magnetic fields [67], Figure 8.

## 3. Scientific Output and User Statistics

### 3.1. Overload Factors

**Figure 9:**
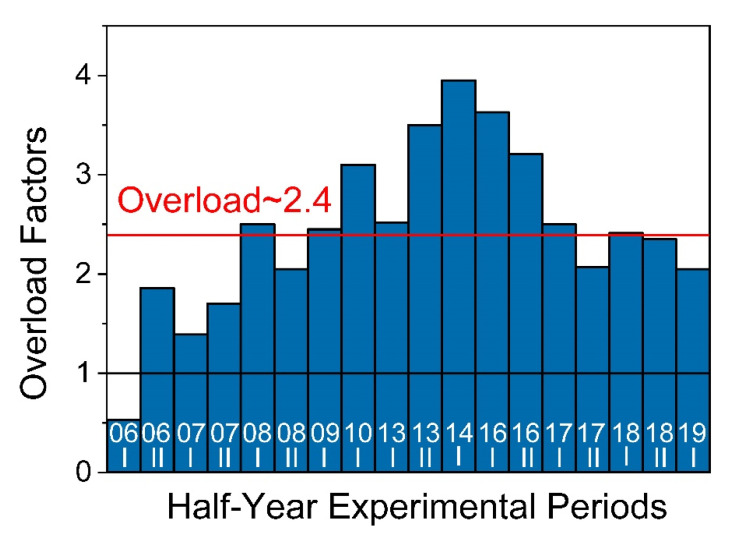
Diagram of the half-year overload factors representing the ratio of requested to available experimental days at the instrument.

For 10 years of operation (without counting the years of reactor shutdowns and instrument upgrades), experiments from 238 accepted proposals were performed at the instrument CONRAD-1/2. The ratios of accepted to requested experimental days per half year, known as overload factors, were calculated and are presented in Figure 9, resulting in an average overload factor of 2.4.

### 3.2. Instrument Profile and User Statistics

The topics of the proposals could be subdivided in the following main groups:

- Material sciences: investigation of morphology and phase transition in metals, like hydrogen embrittlement and austenitic-martensitic phase transition in steels and 3D mapping of cracking and pore distribution in metals, glasses and metallic foam samples.
- Energy sciences: in-situ and ex-situ investigation of dynamic processes in fuel cells, batteries and hydrogen storage materials.
- Geo sciences: water and oil imbibition in rocks, crack propagation and morphological changes in geological samples.
- Life science: plant physiology and soil-root interaction, bone implants and exchange mechanisms in bones and teeth.
- Cultural heritage: investigation of ancient statues, medieval swords and armor attributes, ancient bronze statues and metallic artefacts and paleontological samples from the collection of the Museum of Natural History Berlin.
- Magnetism: fundamental research in the fields of superconductivity and phase transitions in magnetic materials.

**Figure 10:**
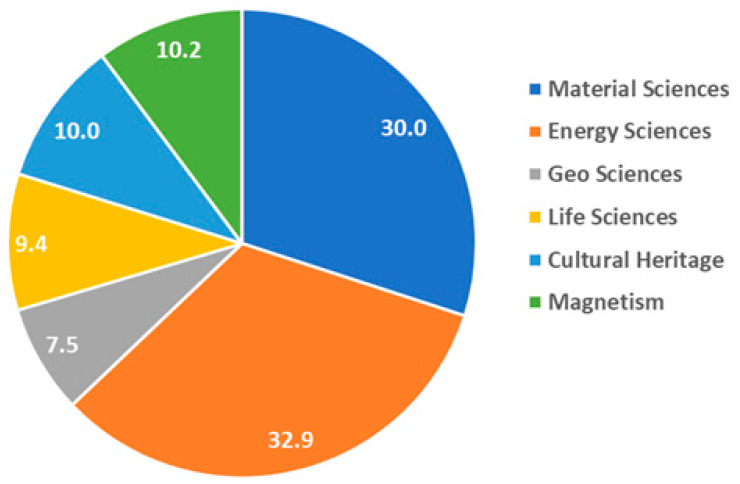
Percentual distribution of the experimental time between the different topics of the accepted proposals.

The distribution of the experimental time between the different topics is shown in Figure 10.

**Figure 11:**
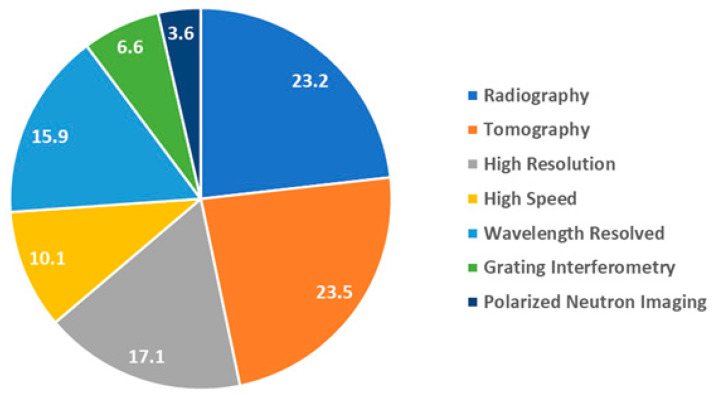
Percentual distribution of the experimental time between the different methods used at the neutron imaging beamline CONRAD-1/2.

For each proposal, the suitable experimental technique was selected in order to obtain the best possible result, Figure 11. The following techniques were available at the CONRAD-1/2 instrument:

- Radiography: observation of dynamic process with moderate time and spatial resolutions (e.g., exposure of seconds and pixel size larger than 20 µm) by recording of 2D transmission images of the sample.
- Tomography: recording of 2D angular projections of the sample with moderate time and spatial resolutions (e.g., exposure of seconds and pixel size larger than 20 µm) and subsequent reconstruction of the 3D tomographic volume using a filtered back-projection algorithm.
- High-resolution: using a high-resolution detector system with pixel size less than 20 µm and thin Gadox scintillator (less than 20 µm).
- High-speed: using a high-speed camera and optimized detector system (200 µm ^6^LiZnS scintillator and light efficient lens system) resulting in exposures of 50–100 ms enabling on-the-fly tomography experiments with bellow one-minute temporal resolution.
- Wavelength-resolved imaging: using the double-crystal monochromator or the velocity selector devices to select a certain neutron wavelength in the range from 1.5 Å to 6.0 Å or to perform a wavelength scan with small steps of typically 0.02 Å for Bragg-edge mapping or contrast enhancement.
- Grating interferometry: using the Talbot-Lau grating interferometry setup in order to perform dark-field or phase-contrast imaging experiments for visualization of magnetic domain walls in electric steels or porosity in additively manufactured metal samples.
- Polarized neutron imaging: using polarizer-analyzer arrangement based on solid state benders for recording the contrast produced by the spin precession of polarized neutron in external or intrinsic magnetic fields.

**Figure 12:**
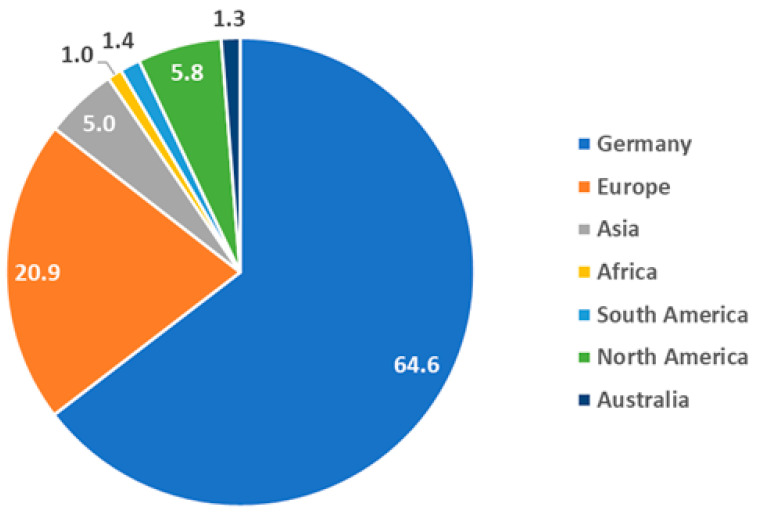
Percentual distribution of the experimental time with respect to geographic base of the principal investigator (PI) proposers.

The distribution of beamtime with regards to the institutional geographic origin of the principal investigator (PI) associated to a beamtime proposal (Figure 12) shows that the CONRAD instrument was predominantly a national facility with a significant orientation to European users mostly from Italy, the UK and Sweden. Asian proposals were mostly from China, North American from US, and South American from Brazil. A few proposals from Africa (South Africa) and Australia (ANSTO) were submitted and accepted as well.

### 3.3. Scientific Output

For the time of its operation, the CONRAD-1/2 instrument produced 211 papers in peer-reviewed journals from 238 accepted proposals, which means that 89% of the principal investigators (PI) published a paper related to the performed experiments. For the time interval of 15 years, this reflects an average number of 14.1 papers per year with 17% of them having a very high-impact factor (IF > 7) and 24% of them having a high-impact factor (7 > IF > 3). A detailed paper statistic is presented in Table 2.

## 4. Conclusions

○ The neutron imaging instruments CONRAD-1/2 served a broad user community from 2005 to the end of 2019, which is reflected in a large number of publications with high scientific as well as societal impact.
○ The improved spatial and temporal resolution capabilities of the instrument, together with the developed and implemented innovative experimental methods including wavelength-selective, dark-field, phase-contrast and polarized neutron imaging, allowed for unique experiments in different scientific fields. Scientific highlights produced by the CONRAD-1/2 instrument are related in particular to polarized neutron imaging, dark-field tomography, wavelength-selective imaging, high-resolution neutron imaging and complementary use of X-ray tomography.
○ The CONRAD-2 instrument stopped its operation due to the shutdown of the research reactor BER II on 11 December 2019.
○ Scientific know-how and advanced hardware will be transferred to the Institute Max Von Laue Paul Langevin (ILL), Grenoble, France in the frame of the Joint Research Unit Ni-Matters.

## Figures and Tables

**Table 1:** Instrument Specifications and Options of CONRAD-2 beamline.

Neutron Guide	NL-1A (m = 2,3) with Beam Cross-Section 125 mm (Height) × 30 mm (Width) Radius of Curvature 750 m
Pinhole changer	1 cm, 2 cm and 3 cm in diameter
Flight path	10 m flight path Aluminum containers filled with He
Measurement positions	Position 1 (end of the guide): Flux: 2.6 × 10^9^ n/cm^2^s @ L/D ca. 70; beam size: 12 × 3 cm Position 2 (5 m from the pinhole): Flux: 7.2 × 10^7^ n/cm^2^s @ L/D 170; beam size: 15 × 15 cm Position 3 (10 m from the pinhole): Flux: 2.4 × 10^7^ n/cm^2^s @ L/D 350; beam size: 30 × 30 cm
Double crystal monochromator	Pyrolytic graphite (002) with mosaicity of 0.8° Wavelength resolution 1–3% Wavelength range: 1.5 Å–6.0 Å
Velocity selector	Wavelength range: 3.0 Å–6.0 Å Wavelength resolution 10–20%
Polarizers	2× Solid-state benders 4× Polarized ^3^He cells and 2× magic boxes
Detectors	CCD camera (Andor, 2048 × 2048 pixels) sCMOS camera (Andor Neo)
Sample positioning	Rotation table (s): 0–360° Translation table: 0–800 mm Lift table: 0–250 mm Goniometer (s): ±20° Maximum weight: 200 kg
Media connections	Cooling water (15 °C), pressurized air (up to 10 bar), nitrogen gas, helium gas, exhaust pipeline. Hydrogen supply system including safety storage box for the bottles, hydrogen sensors, magnetic valve and under-pressure exhaust pipeline.
µ-CT scanner	Micro focus X-ray tube 150 kV (Hamamatsu, L8121-03) and flat panel sensor (Hamamatsu, C7942SK-05) with 2316 × 2316 pixels and a pixel size of 50 µm; cone beam with maximal magnification of 10×.

**Table 2:** Detailed publication statistics of the CONRAD beamline.

Year	Publications	IF > 7	7 > IF > 3	IF < 3
2020	8	2	5	1
2019	20	6	8	6
2018	16	5	5	6
2017	16	2	6	8
2016	12	5	4	3
2015	25	3	4	18
2014	7	2	1	4
2013	7	1	3	3
2012	14	1	1	12
2011	21	4	3	14
2010	15	2	1	12
2009	16	0	3	13
2008	22	3	3	16
2007	4	0	3	1
2006	7	0	1	6
Average	14.1	2.4 (17%)	3.4 (24%)	8.3 (59%)

## Data Availability

Data available in a publicity accessible repository.
